# Cytokine-Like Protein 1(Cytl1): A Potential Molecular Mediator in Embryo Implantation

**DOI:** 10.1371/journal.pone.0147424

**Published:** 2016-01-22

**Authors:** Zhichao Ai, Wei Jing, Li Fang

**Affiliations:** Department of Immunology, Dalian Medical University, Dalian, Liaoning, China; Qingdao Agricultural University, CHINA

## Abstract

Cytokine-like protein 1 (Cytl1), originally described as a protein expressed in CD34+ cells, was recently identified as a functional secreted protein involved in chondrogenesis and cartilage development. However, our knowledge of Cytl1 is still limited. Here, we determined the Cytl1 expression pattern regulated by ovarian hormones at both the mRNA and protein levels. We found that the endometrial expression of Cytl1 in mice was low before or on the first day of gestation, significantly increased during embryo implantation, and then decreased at the end of implantation. We investigated the effects of Cytl1 on endometrial cell proliferation, and the effects on the secretion of leukemia inhibitory factor (LIF) and heparin-binding epidermal growth factor (HB-EGF). We also explored the effect of Cytl1 on endometrial adhesion properties in cell-cell adhesion assays. Our findings demonstrated that Cytl1 is an ovarian hormone-dependent protein expressed in the endometrium that enhances the proliferation of HEC-1-A and RL95-2 cells, stimulates endometrial secretion of LIF and HB-EGF, and enhances the adhesion of HEC-1-A and RL95-2 cells to JAR spheroids. This study suggests that Cytl1 plays an active role in the regulation of embryo implantation.

## Introduction

Embryo implantation in the uterine endometrium is of critical importance for mammalian reproduction and the establishment of pregnancy. This process can be divided into three key stages: apposition, adhesion and invasion[1]. The trophoblastic cells of the blastocyst adhere to the uterine epithelium during apposition and thus, the blastocyst is intimately connected to the receptive epithelium, which facilitates placenta formation. The placenta functions as an interface between the developing embryo and the maternal circulation[2,3]. The success of embryo implantation depends on the temporal and spatial synchronization of adequate cross-talk between the embryo and the uterine epithelium. In humans and other mammals, abundant cytokines and growth factors are involved in a complex sequence of signaling events during implantation. These signals converge around the actions of factors such as interleukin-1 (IL-1), heparin-binding epidermal growth factor (HB-EGF), leukemia inhibitory factor (LIF)[4]. These interactions lead to decidualization, receptive endometrial changes, successful embryo implantation and blastocyst differentiation.

Cytl1, originally cloned from CD34+ human bone marrow or cord blood mononuclear cells[5], was first identified to function as a potential autocrine/paracrine regulatory factor during mesenchymal cell chondrogenesis in vitro[6]. A study of Cytl1 knockout (Cytl1-/-) mice demonstrated that Cytl1 maintains cartilage homeostasis, and deletion of the *Cytl1* gene results in deterioration of osteoarthritic cartilage[7]. Until recently, our knowledge of Cylt1 has been limited to its impact on cartilage development. In immune homeostasis, Cytl1 prevents the development of early-stage inflammatory arthritis and is associated with joint destruction but not with disease progression[8]. Detailed sequence- and structure-based analyses revealed that Cytl1 adopts a 4-helical cytokine structure and IL-8-like chemokine fold templates, similar to the structure of the chemokine (C-C motif) ligand 2 (CCL2), which signals through the CCR2 chemokine receptor[9]. Another report describing the effects of Cytl1 on the growth and metastasis of neuroblastoma (NB) cells revealed a possible relationship between Cylt1 expression and NB development[6]. However, there are no reports describing the role of Cytl1 in embryo implantation. Hence in this study, our aim was to investigate Cytl1 expression patterns both *in vitro* and *in vivo*, and to determine its regulatory effects on the process of embryo implantation.

## Materials and Methods

### Chemical reagents

Recombinant human Cytl1 (donated by the School of Basic Medical Science, Peking University) was dissolved in 100% ethanol to prepare a stock solution of 360 ng/μl and further diluted to final concentrations ranging from 0.1 ng/ml to 10 ng/ml[6] using appropriate cell culture media. Progesterone and estradiol (Sigma, Louis, MO, USA) was dissolved to prepare a stock solution of 0.5 mM and used at final concentrations ranging 10 μM to 50 μM. Anti-Cytl1 antibody (Abcam-129767 Cambridge, UK) was used at a final concentration 1 μg/ml.

### Ethics statement

This study was approved by and conducted according to the guidelines of the Institutional Laboratory Animal Care and Use Committee of Dalian Medical University, China.

### Animals and tissue collection

Male and female Kunming mice (aged 8 weeks, 20–25 g) (n = 30) were purchased from the Specific-Pathogen-Free (SPF) Animal Center of Dalian Medical University (DLM, Dalian, China). Males and females were housed separately in standard cages (4 per cage) at room temperature (22±3°C), with 12 h dark/12 h light cycles and allowed free access to food and clean water. All animal experiments were approved by the Animal Care and Use Committee of DLM according to the Guidelines of Dalian University for the Care and Use of Laboratory Animals. Females were mated individually with adult fertile males each night, and the confirmed mated females were separated. The morning of sighting a vaginal plug was defined as day 0.5 of the pregnancy. Females were euthanized by cervical dislocation, and the endometrium from the control group and pregnant mice was collected on days 0.5, 1.5, 4.5, 6.5 and 8.5, respectively, and stored in RNA protector (Takara Bio, Otsu, Japan) at -80°C. Later, total RNAs were extracted for Cylt1 expression analysis.

### Cell lines and cell culture

Human endometrial cancer cell lines, HEC-1-A (TCHu149) and RL95-2 (TCHu198) and human trophoblastic cancer cell line, JAR (TCHu156) were purchased from the Cell Bank of the Chinese Academy of Science (CAS, China) and were cultured in media according to manufacturer’s protocol. HEC-1-A was maintained in McCoy's 5a medium (Gibco, Carlsbad, CA, USA) supplemented with 10% fetal bovine serum (FBS) (Gibco). RL95-2 was maintained in Dulbecco’s modified Eagle’s medium (DMEM, Gibco)/F-12 (2:3) supplemented with 0.005 mg/ml insulin, 10% FBS. JAR was maintained in DMEM supplemented with 10% FBS. A final concentration of 50 μg/ml streptomycin, and 50 units/ml penicillin was supplemented into each medium. All cells were cultured at 37°C in a humidified atmosphere containing 5% CO_2_. After culturing, the cells or the cell culture supernatants were harvested according to the requirement of experiment.

### RT-PCR and real-time PCR

Total RNA was isolated from the cultured cells and endometrium using RNAiso Plus (Takara, Dalian, China) and the concentration of total RNA was quantified by measuring the absorbance at 260 nm. Reverse-transcription was performed using a PrimeScript^™^ II 1st Strand cDNA Synthesis Kit (Takara Bio) according to the manufacturer’s instructions. RT-PCR was performed using Power Taq PCR MasterMix (Bioteke, Beijing, China). The amplified PCR products of each gene were analyzed by electrophoresis on 1.5% agarose gels. For real-time PCR, cDNA was mixed with primers and SYBR^®^ Premix Ex Taq^™^ II (Takara Bio) and measured on an Mx3000P system (Agilent, StrataGene, USA). β-Actin was amplified as an internal control. For each sample, the relative quantity of each gene was calculated using the 2-^ΔΔ^Ct method[10]. The sequences of primers used for PCR (Table 1) were analyzed using the Basic Local Alignment Search Tool (BLAST) available on the website of the National Center for Biotechnology Information (http://blast.ncbi.nlm.nih.gov/).

**Table 1:** Primers used to amplify target genes in this study.

Gene	Primer sequence	Product length (bp)
Cytl1 (mouse)	F-5' TAGCCTGGCCTCTGGCAGTA 3'	302
	R-5' GAGGAATACCAAGTCCCGCC 3'	
Cytl1 (human)	F-5'AGATCACCCGCGACTTCA 3'	277
	R-5'GTAGTCACTGGGATTGGGTATT 3'	
β-actin (mouse)	F-5' CCAGAGCAAGAGAGGCATCC 3'	226
	R-5' CAACTGTCTCCATGTCGTCC3'	
β-actin (human)	F-5' TCATCACCATTGGCAATGAG3'	155
	R-5' CACTGTGTTGGCGTACAGGT 3'	
LIF (human)	F-5'CCCATCACCCCTGTCAACG3'	176
	R-5'GGGCCACATAGCTTGTCCA3'	
HB-EGF (human)	F-5'TGACCACACAACCATCCTGG3'	276
	R-5'ACTGGGGACGAAGGAGTCTT3'	
CD82 (human)	F-5'TCAGCCTGTATCAAAGTCACC3'	276
	R-5'ATCAGGAGCAGGAAAGCAAAG3'	

### Enzyme-linked immunosorbent assay (ELISA)

The levels of Cytl1 in HEC-1-A, RL95-2 and JAR culture supernatants were analyzed using an indirect ELISA protocol. Briefly, 100 μl of PBS control, positive control and samples were incubated in 96-well plates sealed with adhesive plastic lids overnight at 4°C. Subsequently, 100 μl of anti-CYTL1 antibody(ab129767) used in the dilution of 1:40000 was added (according to the manufacturer’s instructions) and the plates were incubated at 37°C for 2 h. A horseradish peroxidase (HRP)-conjugated diluted secondary antibody (Goat anti-rabbit secondary IgG, HRT Conjugate(Transgen, Beijing,China) used in the dilution of 1:5000 was added and the plates were incubated at 37°C for 2 h. After addition of a substrate (TMB substrate) solution into each well, the reaction was terminated with a stopping solution (2M H_2_SO_4_). After vigorous shaking, the optical density at 450 nm (OD_450_) was measured in least three replicates using a microplate reader(Thermo Fisher Scientific, New York, American).

The levels of LIF and HB-EGF in HEC-1-A, or RL95-2 culture supernatants were quantified with ELISA kits according to the manufacturer’s instructions (Shanghai Lengton Biotechnology, Shanghai, China). Briefly, 50 μl of the PBS control, positive control and samples each were added to 96-well plates. Then after addition of a competitive LIF or HB-EGF detection antigen (Lengton Bio, Shanghai, China), the plates were incubated at 37°C for 1 h, and then washed by a wash buffer. After the addition of substrate and a stopping solution, O.D_450_ was measured in at least three replicates using the microplate reader to verify the reliability of the levels of both LIF and HB-EGF.

### Cell counting kit-8 (CCK-8) assay

Proliferation of HEC-1-A and RL95-2 cells was determined using the CCK-8 assay (Dojindo, Kumamoto, Japan) according to the manufacturer’s instructions. A total of 2,500 cells were seeded into each well in the 96-well plates and allowed to attach overnight before each treatment (described previously). After treatment for 16 or 32 h, the culture supernatants were discarded and 10 μl of a CCK-8 solution was added to each well and incubated at 37°C for 2 h. The OD _450_ and OD _620_ values were measured using the microplate reader. The proliferation of cells was defined as (OD_450_—OD_620)_. At least three independent experiments were performed to verify the reliability of the results, and the time of incubation after treatment was determined in preliminary experiments (data not shown).

### Cell-cell adhesion assay

HEC-1-A and RL95-2 cells were seeded into culture dishes, and incubated at 37°C until the cells reached 90% confluence forming an endometrial cell monolayers. After the addition of JAR cells, the monolayers were treated as described previously and incubated until the adhesive rate of JAR spheroids was measured by a centrifugal force-based adhesion assay. Briefly, JAR spheroids were harvested, counted and seeded onto endometrial cell (HEC-1-A, RL95-2) monolayers. After incubation for 1 h, JAR spheroid adhesion was quantified by centrifuging the dishes (with the bottom facing down) at 12 ×*g* for 5 min. The attached JAR spheroids were counted by CCK-8 quantitation (http://www.dojindo.cn/) and expressed as a percentage of the number of total JAR spheroids seeded. At least three independent experiments were performed to verify the reliability of the results.

### Statistical analysis

The data were expressed as the mean ±SEM, and analyzed using SPSS 19.0 (IBM Corporation, Armonk, NY, USA). The difference between experimental and control groups were calculated using one-way analysis of variance (ANOVA), followed in cases where a significant difference was determined, by the appropriate post-hoc comparison. *P* < 0.05 was considered to indicate statistical significance.

## Results

### Cytl1 is only expressed in endometrial cell lines and predominately expressed in the endometrium during embryo implantation

**Fig 1:**
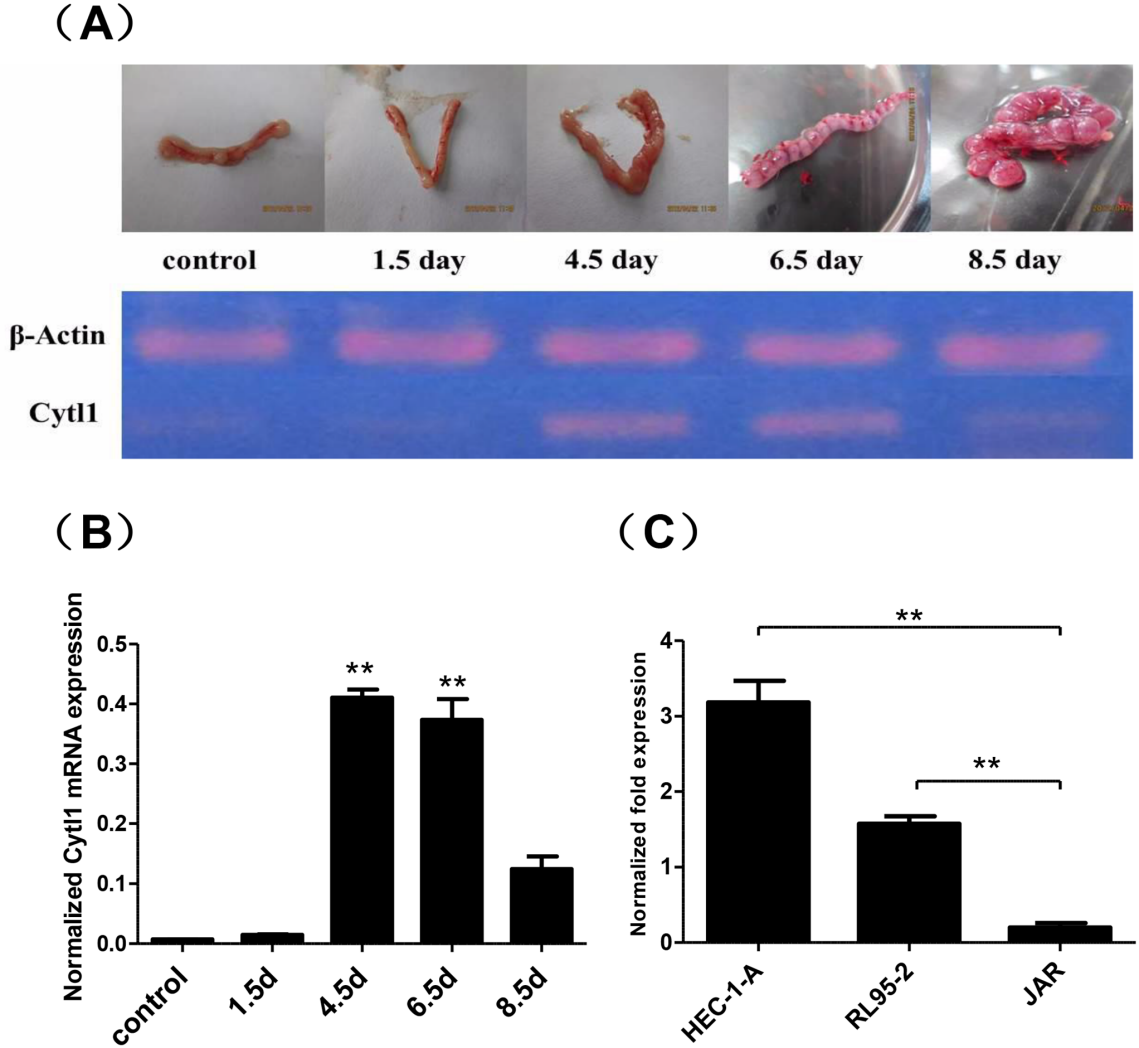
Expression of Cytl1 in mouse endometrial tissues and human endometrial cells. (**A**) Reverse-transcription PCR analysis of Cytl1 mRNA expression in the endometrium at different stages of early pregnancy; representative images of tissues are shown. (**B**) Greyscale analysis of Cytl1 expression relative to β-actin expression. (**C**) Real-time PCR analysis of Cytl1 mRNA expression in human endometrial cell lines (HEC-1-A, RL95-2) and a human trophoblastic cancer cell line (JAR). Data represent mean±SEM. *P < 0.05; **P < 0.01.

RT-PCR analysis suggested that Cytl1 was highly expressed on day 4.5 and day 6.5 of gestation (Fig 1A), which was defined as the period of embryo implantation in mice. In vitro evaluation revealed expression of Cytl1 only in endometrial cell lines, but not in trophoblastic cell lines, suggesting a potential role of endometrial Cytl1 expression during embryo implantation (Fig 1C).

### Ovarian hormones regulate Cytl1 expression at the mRNA and protein levels

**Fig 2:**
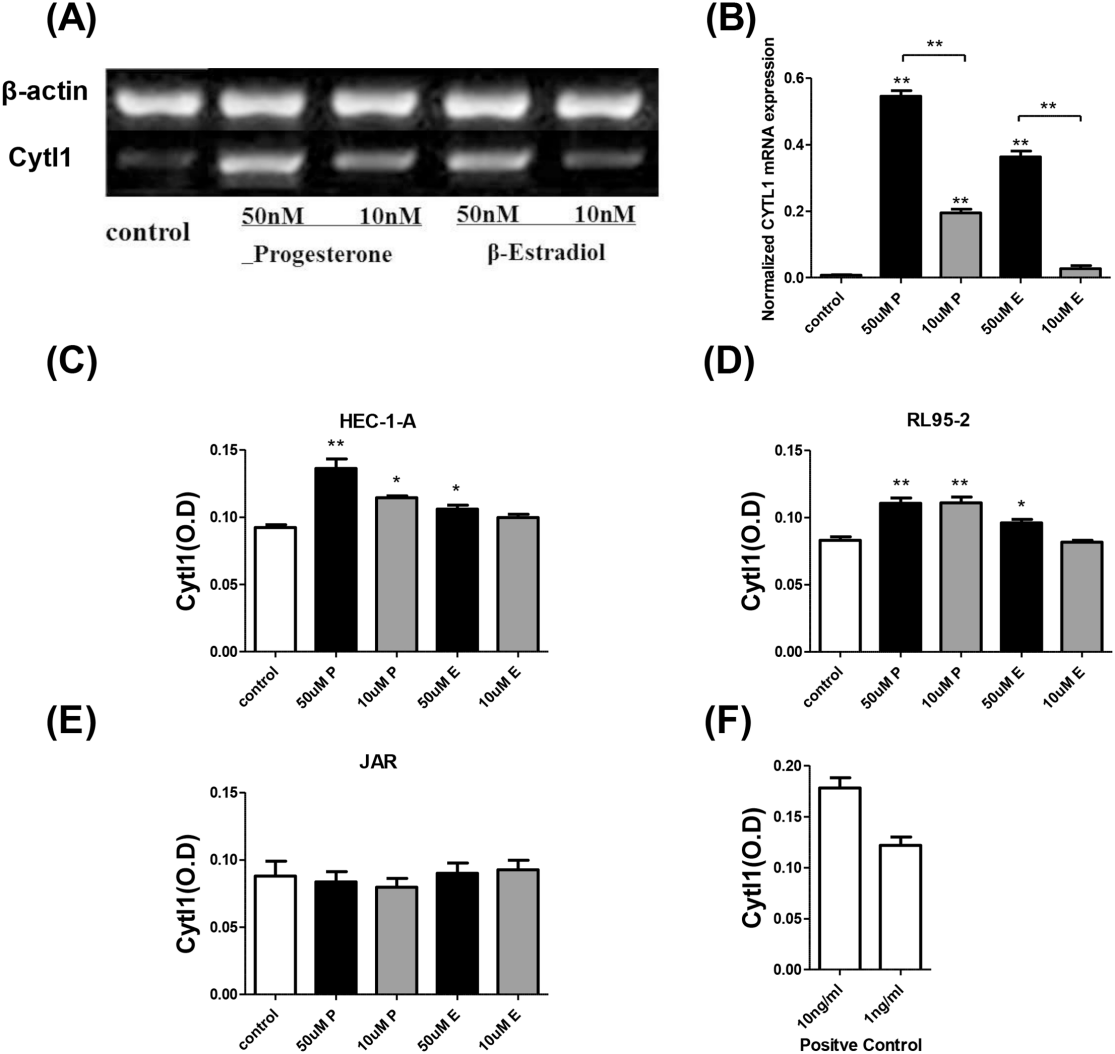
The effects of ovarian hormones on endometrial Cytl1 expression. (**A**) Reverse-transcription PCR analysis of Cytl1 mRNA expression in vitro after treatment with ovarian hormones or control (**B**). Greyscale analysis of Cytl1 expression relative to β-actin expression. Cytl1 protein expression regulated by ovarian hormones or control were analyzed by ELISA in human endometrial cell lines HEC-1-A (**C**), RL95-2 (**D**) and the human trophoblastic cell line JAR. (**E**) Cytl1 expression levels are represented by OD_450_ values, and (**F**) the positive control represents the result of standard sample detected by ELISA. Data shown for each sample are average of results from three array wells. P, progesterone; E, estradiol. Data represent mean±SEM. *P < 0.05; **P < 0.01.

Ovarian hormones regulate the development of the endometrium. After the addition of progesterone or estradiol, Cytl1 expression was detected at the mRNA level by RT-PCR, and at the protein level by ELISA (Fig 2). Cytl1 expression (mRNA and protein) in HEC-1-A and RL95-2 cells was significantly enhanced by the application of 50 or 10 μM progesterone (10 μM or 50 μM) or estradiol (50 μM) compared with the levels detected in the control group (*P* < 0.05), although the levels expressed in JAR cells were almost undetectable. Hence, the concomitant upregulation of Cytl1 and development of the endometrium suggests that Cytl1 is a necessary molecular mediator of peri-implantatory endometrial changes.

### Cytl1 induces endometrial cell proliferation

**Fig 3:**
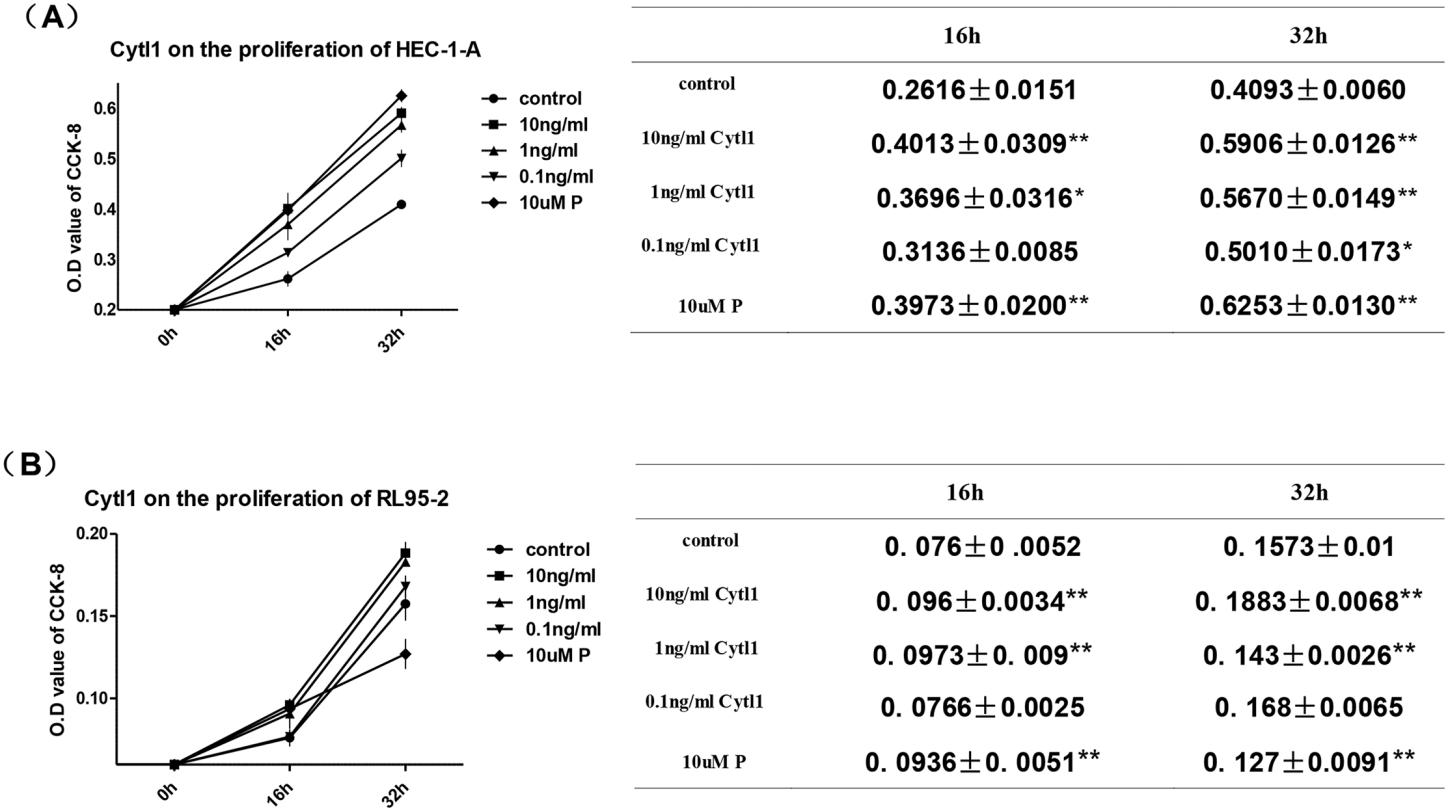
Effects of Cytl1 on endometrial cell proliferation. Proliferation of HEC-1-A (**A**) and RL95-2 (**B**) cells after treatment with different concentrations of Cytl1 or control were determined by CCK-8 assay. Proliferation is represented by optical density (OD) values. P, progesterone. Data represent mean±SEM. *P < 0.05; **P < 0.01.

To determine the role of Cytl1 during endometrial development, we examined the effects of Cytl1 endometrial cell proliferation, and investigated the effects of different concentrations of Cytl1 on HEC-1-A and RL95-2. The addition of Cytl1 (1 ng/ml or 10 ng.ml) significantly promoted the proliferation of HEC-1-A and RL95-2 cells (*P* < 0.05) (Fig 3). Progesterone had a similar effect, which indicates that the endometrial expression of Cytl1 induces endometrial cell growth.

### Cytl1 enhances endometrial cell expression of both LIF and HB-EGF at the mRNA and protein levels

**Fig 4:**
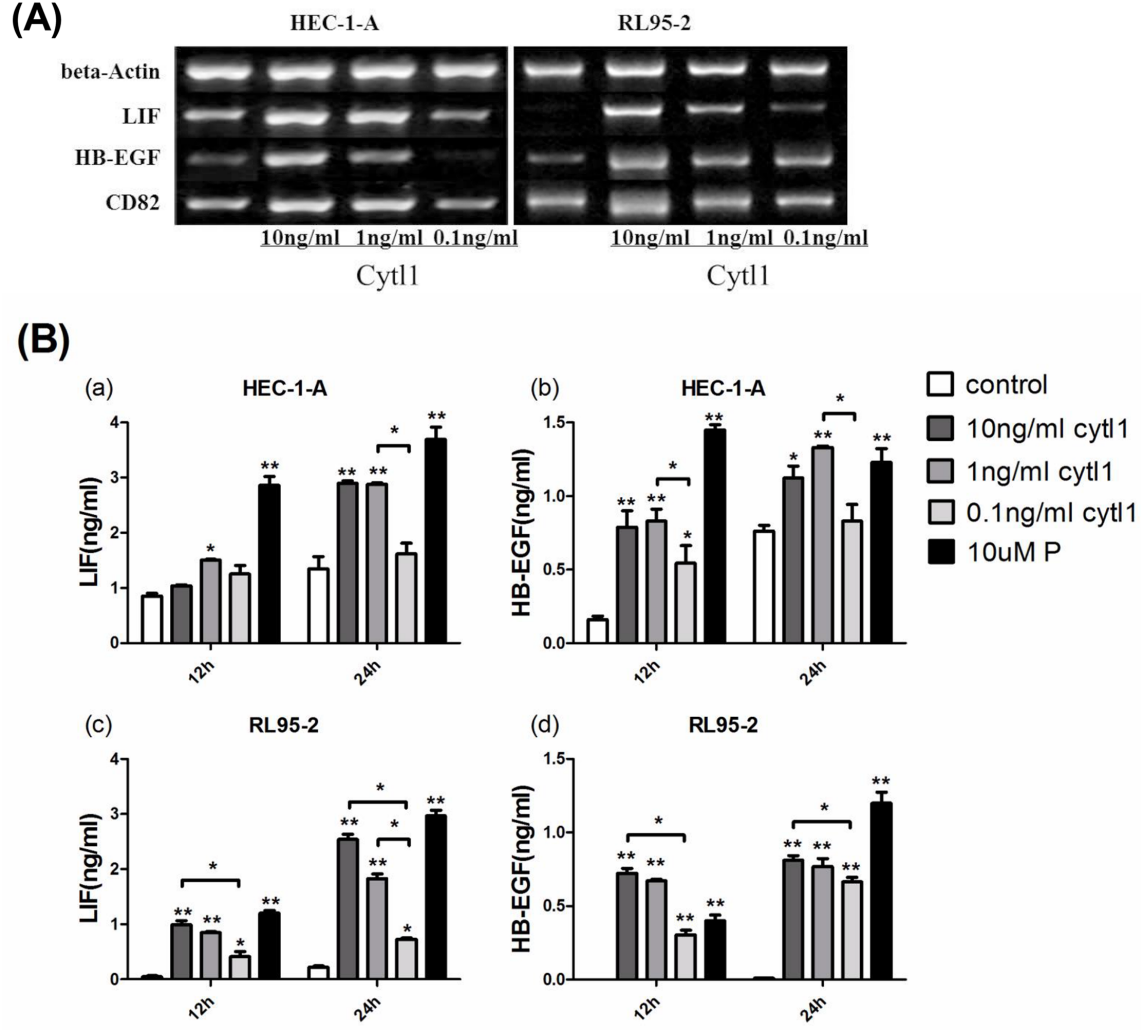
The effect of Cytl1 on the endometrial expression of LIF and HB-EGF. (**A**) Reverse -transcription PCR analysis of LIF and HB-EGF mRNA expressions in human endometrial cell lines (HEC-1-A, RL95-2) after treatment with different concentrations of Cytl1. LIF expression in the culture supernatant of HEC-1-A (**Ba**) and RL95-2 (**Bc**), and HB-EGF expression in HEC-1-A (**Bb**) and RL95-2 (**Bd**) were detected by ELISA. The standard curve prepared according to manufacturer’s instruction was used to determine sample concentrations. P, progesterone. Data represent mean±SEM. *P < 0.05; **P < 0.01.

LIF and HB-EGF were chosen as the regulatory indicators of implantation to evaluate the role of Cytl1 in early pregnancy. The levels of the LIF, HB-EGF and CD82 mRNA expression in HEC-1-A and RL95-2 cells significantly increased after the application of Cytl1 at 1 ng/ml or 10 ng/ml (*P* < 0.05), but had no effects at 0.1 ng/ml (*P* > 0.05) (Fig 4A). Higher concentrations of LIF and HB-EGF were detected in HEC-1-A and RL95-2 culture supernatants under the influence of Cytl1 (*P* < 0.05) (Fig 4B). Briefly, the application of Cytl1 significantly enhanced endometrial expression of LIF and HB-EGF at both the mRNA and protein levels, which indicates its significant effects during embryo implantation.

### Cytl1 enhances the endometrial cell adhesion to trophoblastic cells

**Fig 5:**
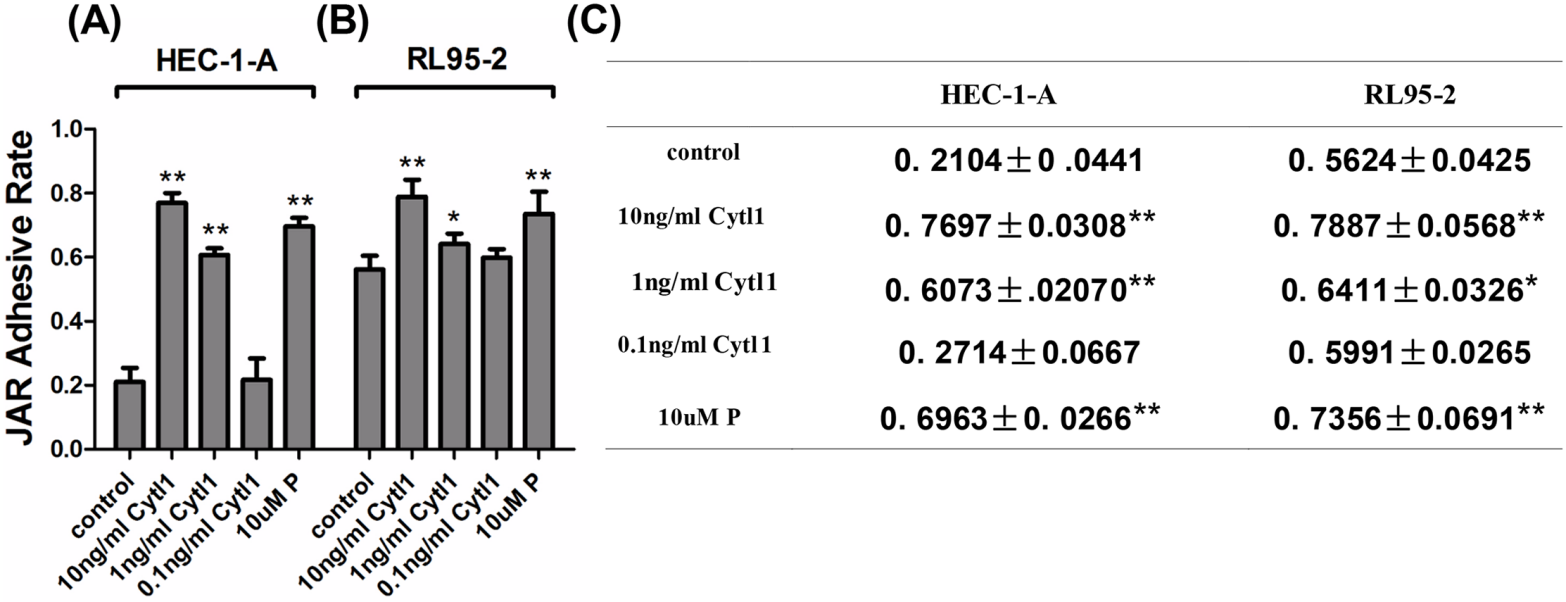
The effect of Cytl1 on JAR spheroid adhesion to endometrial cells. The percentages of JAR spheroid adhesion to HEC-1-A (**A**) and RL95-2 (**B**) cells were determined using cell-cell adhesion assays; JAR spheroid adhesion was quantified using CCK8 assays; results are presented as optical density (OD) (**C**). P, progesterone. Data represent mean±SEM of three independent experiments. *P < 0.05; **P < 0.01.

After incubation in the presence of Cytl1, the percentage of JAR spheroids adhering to HEC-1-A significantly increased from 21.04±4.41% in the control group to 76.97±3.08% and 60.73±2.07% after the application of 10 ng/ml and 1 ng/ml Cytl1, respectively. The percentage of JAR spheroids adhering to RL95-2 significantly increased from 56.24±4.25% in the control group to 78.87±5.68% and 64.11±3.26% after the application of 10 ng/ml and 1 ng/ml Cytl1, respectively (*P* < 0.05) (Fig 5), which indicates that Cytl1 significantly enhances endometrial cell adhesion to trophoblastic cells.

## Discussion

This is the first study of the potential role of the expression of the functional secreted protein Cytl1 at the key stages in early pregnancy, which includes the development of endometrial receptivity, and the key stages of embryo implantation into the maternal endometrium. Under the influence of estrogen and progesterone, many changes in the endometrium are induced to enhance its receptivity to an implanting blastocyst[11], one of the most important changes being the expression of secreted and membrane-bound signaling factors that influence the apposition, adhesion and invasion of embryo implantation.

In the present study, Cytl1 showed a pattern of endometrial expression that it is barely detectable before or on the first day of pregnancy, followed by sudden and significantly increased expression on day 4.5 and day 6.5 of pregnancy, corresponding to the implantation window of endometrial receptivity and the behavior of embryo implantation[12]. The expression in endometrium was found to be relatively low on day 8.5, suggesting its role in early pregnancy. The success of implantation depends on the temporal and spatial synchronization of cross-talk between blastocyst trophoblasts and the uterine endometrium, which involves a variety of interrelated molecules[13]. Protein sequence analysis revealed that the regulatory effect of Cytl1 is homologous to that of the IL-2-related common gamma chain-dependent cytokines. Recent studies using structure-based analysis techniques indicate that Cytl1 is a structurally and functionally related CCL-2 analog that signals through the CCR2 chemokine receptor, the ligand of which is involved in angiogenesis and macrophage recruitment and activation[14,15]. All these results suggest that Cytl1 plays a role in embryo implantation.

The process of embryo implantation can be divided into three steps: apposition, adhesion and invasion[13]. During blastocyst apposition, trophoblastic cells adhere to a receptive endometrium, the establishment of which is controlled predominately by ovarian hormones. These hormones induce morphological and phenotypic changes in the endometrial cells, and thus promote the synthesis of cytokines and molecular mediators contributing to embryo implantation[16]. The regulation of endometrial Cytl1 expression was found to be subject to positive feedback in our study. Cytl1 expression in endometrial cells was significantly enhanced with increasing concentrations of progesterone and estrogen, implicating Cytl1 as a candidate marker of endometrial receptivity. Further investigation showed that the ovarian hormone-induced upregulation of Cytl1 production led to a significant increase in endometrial cell proliferation.

The trophoblast cells of the blastocyst adhere to the endometrium to establish the interactions between the embryo and maternal uterus. Cytokines function as intermediary links of the early fetal-maternal relationship. Leukemia inhibitor factor (LIF) and heparin-binding EGF-like growth factor (HB-EGF) are among a number of molecular mediators involved in this early fetal-maternal interaction[17,18]. The vital role of LIF during embryo implantation has been established based on abnormal LIF levels in infertile patients and LIF gene mutations in patients with repeated implantation failure[19]. Increased HB-EGF production during both the menstrual cycle and implantation has been observed in the endometrium and evidence supporting the important role of HB-EGF in implantation have been comprehensively reviewed[18,20]. Briefly, both LIF and HB-EGF play key roles in implantation and blastocyst growth. Thus, LIF and HB-EGF were selected and used in further experiments to further elucidate the role of Cytl1 in implantation. Cytl1 (10, 1, and 0.1 ng/ml) stimulated endometrial production of LIF and HB-EGF. The role of Cytl1 as a regulatory factor was suggested by its influence on mesenchymal cell chondrogenesis and neuroblastoma cell growth, as well as its protective effects on cartilage development[6,7]. Our study suggests that Cytl1 induces a remarkable increase in LIF expression, and significantly affected the levels of HB-EGF in cultured endometrial cells. Our results are consistent with those reported by Jeon who demonstrated that Cytl1 functions via stimulating the transcriptional activity of Sox9 and induces the production of insulin-like growth factor (IGF-1), which stimulates the secretion of LIF[21] and HB-EGF[16]. Moreover, cell-cell adhesion assay showed that Cytl1 also significantly enhances the adhesion of endometrial cells to trophoblastic cells, and upregulates the mRNA expression of CD82, a potential adhesive molecule. It can be speculated that Cytl1 regulates the adhesive competence of endometrial cells via the upregulation of LIF[21].

In summary, our study shows that Cytl1 is highly expressed in the endometrium during the embryo implantation process and the Cytl1 expression pattern is regulated by ovarian hormones. Furthermore, we showed that Cytl1 enhances endometrial proliferation, induces the secretion of endometrial LIF and HB-EGF, and even enhances endometrial cell adhesion to trophoblastic cells. All these results indicate that Cytl1 is a potential molecular mediator of embryo implantation although the molecular mechanisms underlying this effect remain to be further elucidated.
